# An Arteriovenous Bioreactor Perfusion System for Physiological In Vitro Culture of Complex Vascularized Tissue Constructs

**DOI:** 10.3390/bioengineering11111147

**Published:** 2024-11-14

**Authors:** Florian Helms, Delia Käding, Thomas Aper, Arjang Ruhparwar, Mathias Wilhelmi

**Affiliations:** 1 Lower Saxony Center for Biomedical Engineering, Implant Research and Development (NIFE), Stadtfelddamm 34, 30625 Hannover, Germany; 2 Division for Cardiothoracic, Transplantation and Vascular Surgery, Hannover Medical School, 30625 Hannover, Germany; 3 Department of Vascular and Endovascular Surgery, St. Bernward Hospital, 31134 Hildesheim, Germany

**Keywords:** bioreactors, perfusion systems, hemodynamic simulators, vascular tissue engineering, regenerative medicine, in vitro models

## Abstract

Background: The generation and perfusion of complex vascularized tissues in vitro requires sophisticated perfusion techniques. For multiscale arteriovenous networks, not only the arterial, but also the venous, biomechanical and biochemical conditions that physiologically exist in the human body must be accurately emulated. For this, we here present a modular arteriovenous perfusion system for the in vitro culture of a multi-scale bioartificial vascular network. Methods: The custom-built perfusion system consisted of two circuits: in the arterial circuit, physiological arterial biomechanical and biochemical conditions were simulated using a modular set-up with a pulsatile peristaltic pump, compliance chambers, and resistors. In the venous circuit, venous conditions were emulated accordingly. In the center of the system, a bioartificial multi-scale vascularized fibrin-based tissue was perfused by both circuits simultaneously under biomimetic arteriovenous conditions. Culture conditions were monitored continuously using a multi-sensor monitoring system. Results: The physiological arterial and venous pressure- and flow-curves, as well as the microvascular arteriovenous oxygen partial pressure gradient, were accurately emulated in the perfusion system. The multi-sensor monitoring system facilitated live monitoring of the respective parameters and data-logging. In a proof-of-concept experiment, vascularized three-dimensional fibrin tissues showed sustained cell viability and homogenous microvessel formation after culture in the perfusion system. Conclusions: The arteriovenous perfusion system facilitated the in vitro culture of a multiscale vascularized tissue under physiological pressure-, flow-, and oxygen-gradient conditions. With that, it presents a promising technique for the in vitro generation and culture of complex large-scale vascularized tissues.

## 1. Introduction

Dynamic bioreactor perfusion systems have gained immense importance in cardiovascular tissue engineering especially for the generation of complex vascularized networks, as well as for in vitro pathogenesis analyses and drug-screening platforms [1,2]. The connection to the vascular system, as well as the implant-internal vascular supply and perfusion take a key role in modern implant research. Any bioartificial tissue or organ exceeding the maximum distance for cell nutrition via passive diffusion obligatorily requires internal perfusion and connection to the vasculature of the implant recipient. Thus, the integration of a vascular network into bioartificial implants using vascular tissue engineering techniques plays a decisive role on two levels: on the one hand, large-diameter vessels must be implemented in bioartificial organs and tissues to enable surgical anastomoses to the arterial and venous system of the implant recipient. On the other hand, a dense microvascular and capillary network is required, which allows a sufficient and homogeneous supply of the functional cells of the biologically active implant. To ensure direct perfusion and cell nutrition immediately after implantation, macrovessels and microvascular capillaries must be interconnected via a hierarchical vascular network, as is also present in native tissues.

For this, both biomechanical and biochemical conditions of the physiological human microcirculation must be taken into account [3,4]. The native microvasculature is exposed to a complex and dynamic biomechanical environment influenced by pulsatile arterial pressure with a physiological dicrotic pressure curve [5], as well as cyclic venous pressure variations [6]. In addition to this dynamic pressure environment resulting in cyclic distension and relaxation especially in larger vessels, flow conditions also vary distinctively between the arterial and venous side of the human microvascular network. In peripheral arteries, a typical three-phasic flow curve can be observed, while peripheral veins show a more or less stationary flow behavior [7]. Moreover, an arteriovenous oxygen gradient is present within the course of the microvasculature ranging from the typical arterial oxygen partial pressure of 80–100 mmHg to venous oxygen partial pressures of 30–50 mmHg.

It has already been shown that accurate perfusion in dynamic bioreactor systems is of pivotal importance both for the generation of macrovessels [8] and for the formation of capillary networks [9,10]. Other groups have shown that perfusion parameters also play an essential role in the regulation of vascular sprouting, i.e., the formation of microvascular side branches from a larger parent vessel [3,11]. For this phenomenon, oxygen gradients have also been shown to promote angiogenesis and sprouting in in vitro models [12].

Consequently, mimicking the physiological biomechanical environment, as well as the oxygen gradient observed in the human microvasculature, is a promising approach for generating vascular networks in vitro. Thus, for the generation of multiscale vascular networks in bioartificial tissues, sophisticated in vitro perfusion systems are essential.

In the recent past, numerous in vitro hemodynamic simulators have been developed by us and others. Existing systems can be divided into two groups: hemodynamic simulators are focused on accurate in vitro pressure and flow simulations and do not facilitate the introduction of biological constructs and are therefore not suitable for tissue engineering approaches [13,14,15]. On the other hand, vascular bioreactor perfusion systems allow for the implementation of native or bioartificial biological probes. However, these systems usually focus on one distinctive part of the vasculature, such as exclusively small-lumen arteries or exclusively capillary networks and are therefore unsuitable to emulate the complex biomechanical environment of the human microvasculature [8,16,17,18,19].

Thus, a comprehensive in vitro perfusion system for the accurate perfusion of arteriovenous vascular constructs is not available to date. Consequently, the aim of this work was to establish an arterio-venous perfusion system, which accurately emulates both the arterial and venous pressure and flow conditions, as well as the physiological arteriovenous oxygen partial pressure gradient, in three-dimensional fibrin-based bioartificial tissue matrices.

## 2. Materials and Methods

### 2.1. Perfusion Chamber Setup

**Figure 1:**
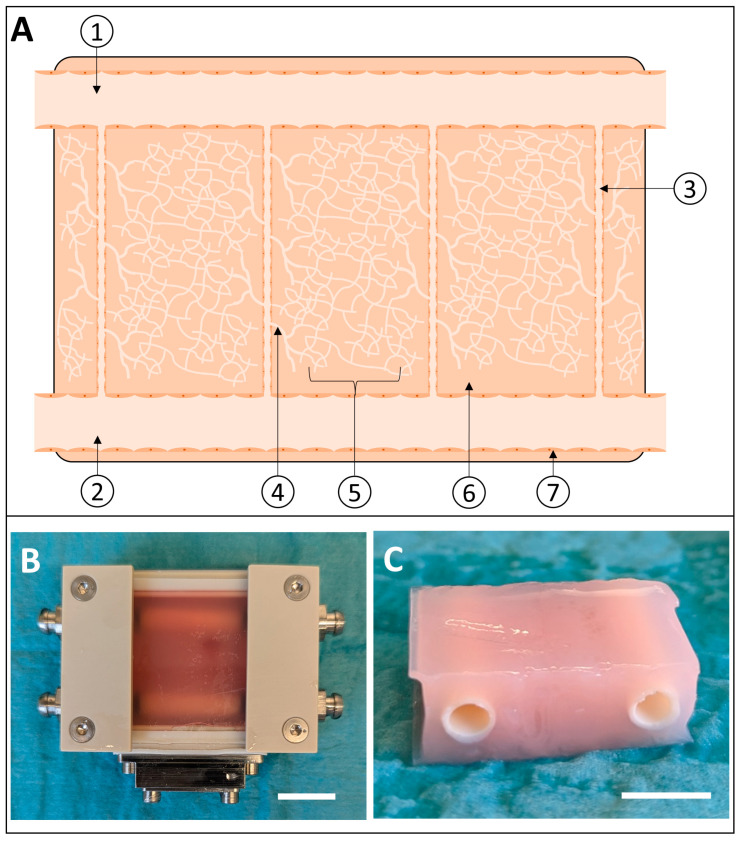
Generation of the vascularized fibrin-based matrix. (**A**) Schematic cross-section of the targeted multi-scale vasculature. Venous and arterial fibrin-based macrovessels (1 + 2) were placed in parallel to each other and interconnected via four microchannels (3). Vascular sprouts arising from the microchannels (4) were intended to interconnect the microchannels to a capillary network built-up by the co-culture of human umbilical vein derived endothelial cells (HUVECs) and adipogenous stem cells (5) seeded throughout a low-density fibrin matrix (6). Both macrovessels and microchannels were enothelialized by a HUVEC monolayer (7). Black arrows indicate the media flow direction during perfusion. (**B**) Perfusion chamber with the integrated fibrin-based tissue construct. Two hose nozzles on each side facilitated connection of the integrated macrovessels to the respective arterial and venous perfusion circuit, and the perforated sheath on the bottom allowed for insertion of needles during the molding process for the generation of the microchannels. (**C**) Macroscopic morphology of the explanted fibrin-based tissue matrix after 48 h of culture in the arteriovenous perfusion system. Scale bar = 1 cm.

A custom-built perfusion chamber was designed and build in cooperation with the department for medical device construction at Hannover Medical School [20]. Briefly, the perfusion chamber used here functioned as both the mold for the generation of the tissue matrix and the bioreactor for subsequent perfusion. It consisted of polyether ether ketone (PEEK) and enclosed a central cavity of 0.5 cm × 3 cm × 3 cm, which defines the dimensions of the fibrin-based tissue matrix. The chamber was closed by a removable glass plate on each side (Figure 1B). Two adapters on each end of the chamber facilitated the integration of the fibrin-based macrovessels. For perfusion, the adaptors had integrated hose nozzles, which allowed the connection of the perfusion tubes in the perfusion system. A perforated metal plate was installed on one side of the perfusion chamber as a sheath allowing the insertion of needle cannulas in a 90° angle to the macrovessels for the generation of the microchannels.

### 2.2. Generation of the Cellularized Fibrin-Based Tissue Matrix

The bioartificial fibrin-based tissue matrices were built up by three components: two fibrin-based macrovessels, which facilitate the connection to the perfusion system, a tissue matrix based on a low-concentration fibrin matrix, which was cellularized with a co-culture of red-fluorescent-protein-expressing human umbilical vein endothelial cells (RFP-HUVEC) and adipogenous stem cells (ASCs), and three microchannels interconnecting the two macrovessels (Figure 1A). The tissue matrices were generated via modification of a previously established protocol [20]. In the first step, the fibrin-based macrovessels were fabricated using a centrifugation technique as previously described [16]. The resulting fibrin-based vessels were cut to a length of 3 cm each. During implementation in the perfusion chamber, the vessels were stretched longitudinally when they were fixated onto the adapters of the flow chamber. Both macrovessels were inserted into the system in parallel to each other. Subsequently, three needle cannulas with an outer diameter of 1.2 mm were inserted through the perforated metal sheath perpendicular to the macrovessels penetrating the arterial macrovessel and the medial wall of the venous macrovessel to generate the connections between both vessels, which will later result in the microchannels. After this step, the perfusion chamber was closed and sealed for the molding of the fibrin matrix containing RFP-HUVECs and ASCs. The latter were harvested from the subcutaneous adipose tissue of a donor scheduled for visceral surgery after informed consent and ethical approval by the ethics commission of Hannover Medical School, as previously described [21]. RFP-HUVECs and ASCs were suspended in a solution containing 10 mg/mL cryoprecipitated fibrinogen replenished in human blood serum, M199 10X (100 µL/mL), and Aprotinin (100 U/mL). The pH was adapted through the titration of 1 N NaOH. During molding, the suspension was mixed with an equal volume of a solution containing Thrombin (2.5 U/mL), Factor XIII (2.5 U/mL), and 40 mM CaCl_2_ in a double-barreled syringe giving a final fibrin concentration of 5 mg/mL. REP-HUVECs and ASCs were seeded at a ratio of 1:0.5 with a final concentration of 1 × 10^6^/mL for the HUVECs and 0.5 × 10^6^ for the ASCs. The perfusion chamber was filled and deaired completely followed by static incubation at room temperature for 30 min to facilitate polymerization of the fibrinogen and formation of the fibrin-based matrix.

### 2.3. Bioreactor Perfusion System Setup

**Figure 2:**
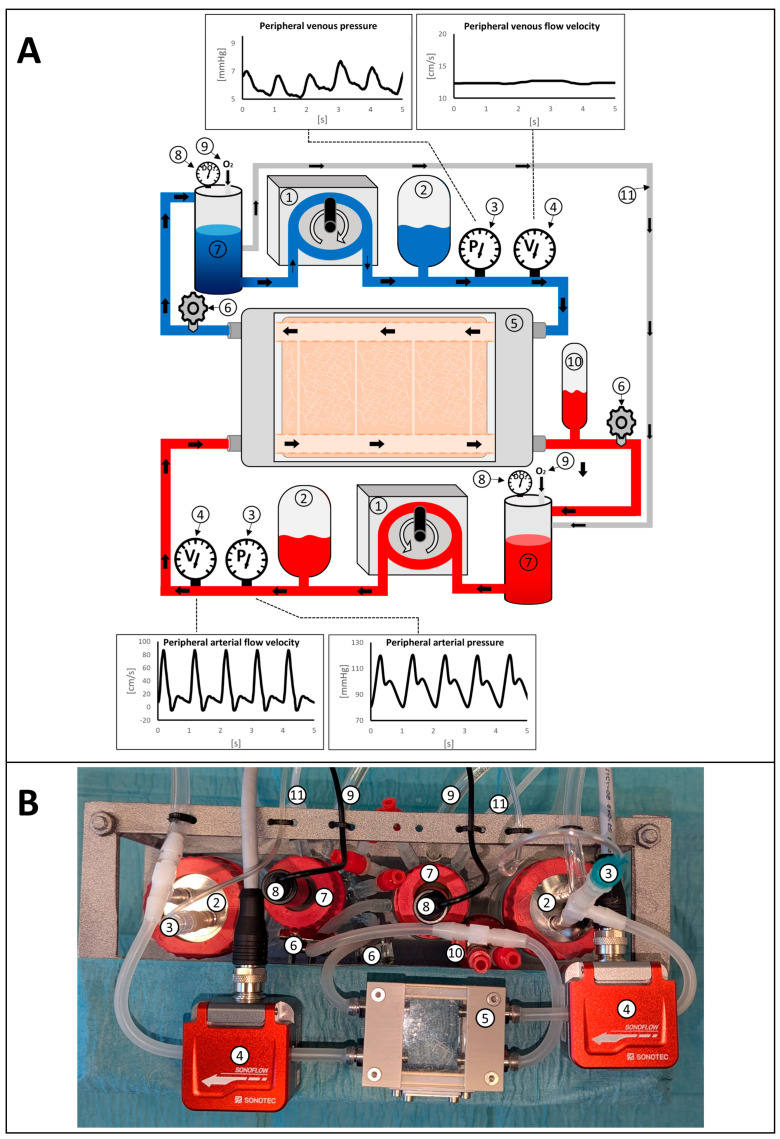
(**A**) Schematic representation of the arteriovenous perfusion system setup and desired pressure and flow curves. 1: Pulsatile peristaltic pump; 2: upstream compliance chamber; 3: pressure sensor; 4: flow sensor; 5: perfusion chamber with the integrated fibrin-based matrix and vessels; 6: variable resistor; 7: reservoir; 8: dissolved oxygen sensor; 9: oxygen inflow canula; 10: downstream arterial compliance chamber; 11: backflow line. (**B**) Photographic top-view of the assembled system.

The bioreactor perfusion system consists of two interconnected circuits (Figure 2): an arterial circuit (red in Figure 2) in which the physiological arterial pressure and flow profile, as well as arterial oxygen saturation, were simulated, and a venous circuit (blue in Figure 2) in which the corresponding parameters typical for the venous vasculature of the human body were mimicked. Both circuits had a similar modular setup. Each circuit consisted of a non-occlusive peristaltic pump equipped with two check valves, one upstream and one downstream of the pump, for directed pulsatile media flow. The rotation speed and the extent of tube compression by the roller pump were adapted to the desired cyclic pressure and flow variations for the arterial and venous system, respectively. Downstream of that, variable compliance chambers were installed as side branches in each circuit to mimic vascular compliance and the “Windkessel”-effect of the aorta in the arterial system. The effectiveness of the impulse damping was adapted through variation of the air/liquid ratio in each chamber. The centerpiece of the perfusion system was the custom-built perfusion chamber. The hose nozzles on which the arterial fibrin vessel was mounted were connected to the arterial circuit of the perfusion system, and the venous fibrin macrovessel was connected to the venous circuit accordingly. Hereby, the two macrovessels were integrated into the perfusion system in a manner that the direction of flow in the venous vessel was opposite to the flow in the arterial vessel within the perfusion chamber. The arterial circuit was equipped with an additional compliance chamber placed as a side branch after the perfusion chamber. This chamber showed significantly lower compliance than the compliance chamber placed upstream of the perfusion bioreactor. With that, the downstream compliance chamber was implemented to facilitate emulation of the typical tri-phasic flow profile with late-systolic flow reversal as present in peripheral arteries in the human vasculature. A variable resistance was placed downstream of the perfusion chamber to facilitate adaption of the targeted arterial and venous mean pressure, as well as modulation of the respective flow rates. Each reservoir was equipped with a separate oxygen inflow canula for regulation of the desired oxygen partial pressure in each circuit. To compensate for the shunt flow from the arterial to the venous circuit via the microchannels, an additional backflow line was installed between the arterial and venous reservoirs.

### 2.4. Monitoring and Data Logging

Pressure, flow, and dissolved oxygen partial pressure were monitored continuously using a custom-built multisensor monitoring system. All sensors were connected to an Arduino^®^ Mega 2560 microcontroller board (Arduino S.r.l., Monza, Italy) for continuous and simultaneous monitoring using a C++ based custom-built program. The software Telemetry Viewer Version 8.0 (Farrell Farahbod) was used for live data visualization and long-term data logging. For pressure monitoring, single axial barbed pressure sensors (Honeywell, Morristown, NJ, USA) with a maximum analog output rate of 1 kHz were implemented directly upstream to the perfusion chamber in each circuit. The flow was monitored in each circuit using ultrasonic clamp-on flow meters (Sonotec^®^, Halle, Germany) with an output rate of 80 Hz. With that, continuous real-time monitoring of the pressure and flow conditions was facilitated. The dissolved oxygen partial pressure was measured using analog dissolved oxygen sensors (DFRobot, Shanghai, China), which were submerged in the media in the reservoirs. Signals from these sensors were used for both monitoring and visualization of the oxygen partial pressures and adaption of the oxygen partial pressures to the desired values. For that, the dissolved oxygen partial pressure was adapted automatically using electric solenoid valves (ACL^®^, Cavenago di Brianza, Italy) controlling the oxygen inflow to each reservoir. The solenoid valves were controlled by the Arduino Mega microcontroller board using a C++-based code. With that, oxygen supply to the system was continuously adapted to the dissolved oxygen partial pressure measured by the respective sensors in reference to the pre-set target oxygen partial pressure for each circuit.

### 2.5. Microchannel Perfusion

In a pretest, the feasibility of the arteriovenous perfusion connecting the arterial and venous circuit via the microchannels penetrating the cellularized fibrin matrix was tested. For this, a cellularized fibrin-based tissue matrix seeded with RFP-HUVECs and ASCs was perfused in the bioreactor system directly through the microchannels for 48 h using the same culture media and culture conditions described below. The shunt flow and perfusion pressure were measured continuously to test for patency of the microchannels. Shunt flow was measured using the clamp-on sonographic flow sensors and the software “Sonotec C3” (Sonotec^®^, Halle, Germany). Perfusion pressures were monitored using axial barbed pressure sensors for each channel.

### 2.6. Perfusion of Cellularized Tissue Constructs

For an initial proof of concept, a cellularized fibrin-based tissue matrix seeded with RFP-HUVECs and ASCs was cultivated in the perfusion system. For this, the system containing the implemented perfusion chamber with the cellularized fibrin matrix was placed in a cell incubator at 37 °C and with carbon dioxide saturation of 5%. As perfusion media, Medium 199 supplemented with 40 ng/mL vascular endothelial growth factor (VEGF), 40 ng/mL fibroblast growth factor (FGF), 50 μg/mL L-ascorbic acid-2-phosphate, and 100 U/mL aprotinin (all from Sigma-Aldrich, Steinheim, Germany) was used, and perfusion was performed for 48 h.

Afterwards, the matrix was fixated in a solution containing 4% paraformaldehyde. For analysis, fluorescence microscopy was performed. For this, 5 mm-thick probes of the matrix were dehydrated in ethanol solutions of 50% for 4 d, 75% for 2 d, and 100% for 4 d. Subsequently, the refractive index was adapted through incubation in methyl salicylate and benzyl benzoate (both from Sigma Aldrich) in a mixing ratio of 5:3 for 24 h. Fluorescence microscopy was performed using the Nicon Eclipse TE300 inverse fluorescence microscope (Nikon, Tokyo, Japan). Networks and crossing points were visualized using the software “AngioTool” Version 0.6 [22] . To further investigate the microanatomy of the capillary-like tubes, transmission electron microscopy was performed. For this, the tissue was fixated in a solution with 1.5% paraformaldehyde (Sigma-Aldrich, Steinheim, Germany), 1.5% glutaraldehyde (Merck, Darmstadt, Germany), and 150 mM HEPES (Sigma-Aldrich) at pH 7.35 for a minimum of 30 min. After sectioning, images were acquired using the FEI Morgagni 268 microscope (FEI, Eindhoven, The Netherlands). In a pre-test, endothelial cell viability was analyzed on cross-sections using a DMEM-based staining solution containing 0.5 µmol/L calcein (Live/Dead viability/cytotoxicity kit, Life Technologies, Darmstadt, Germany). After 48 h of perfusion, sections were incubated in the staining solution for 45 min prior to fluorescence microscopy analysis. For quantification, three random cross sections of different parts of the matrix were analyzed using the software “ImageJ” (Version 1.54i). Viability was defined as the ratio of the number of living, calcein-stained endothelial cells and the total number of endothelial cells visualized based on RFP.

### 2.7. Statistical Analysis

Statistical analysis was performed using the Software “GraphPad Prism 6.04” (GraphPad software, San Diego, CA, USA). Data are given as the mean ± standard deviation.

## 3. Results

### 3.1. Generation of Bioartificial Fibrin-Based Matrices and Bioreactor Setup

Molding of the matrices resulted in constructs measuring 0.5 cm × 3 cm × 3 cm consisting of a low-density fibrin matrix containing the RFP-HUVEC and ASC co-culture for capillary tube formation, penetrating microvessels, and encapsulated high-density fibrin-based macrovessels (Figure 1C). Molding and centrifugation of the macrovessels resulted in an outer diameter of 6.76 mm ± 0.125 mm and an inner diameter of 5.04 mm ± 0.21 mm (*n* = 6). These vessels, when encapsulated in the fibrin-based matrix, provided sufficient biomechanical stability to withstand the dynamic arterial and venous perfusion in the perfusion system. Continuous arteriovenous shunt flow through the microchannels was observed during the perfusion period indicating patency of the microchannels.

### 3.2. Simulation of Physiological Pressure and Flow Conditions

**Figure 3:**
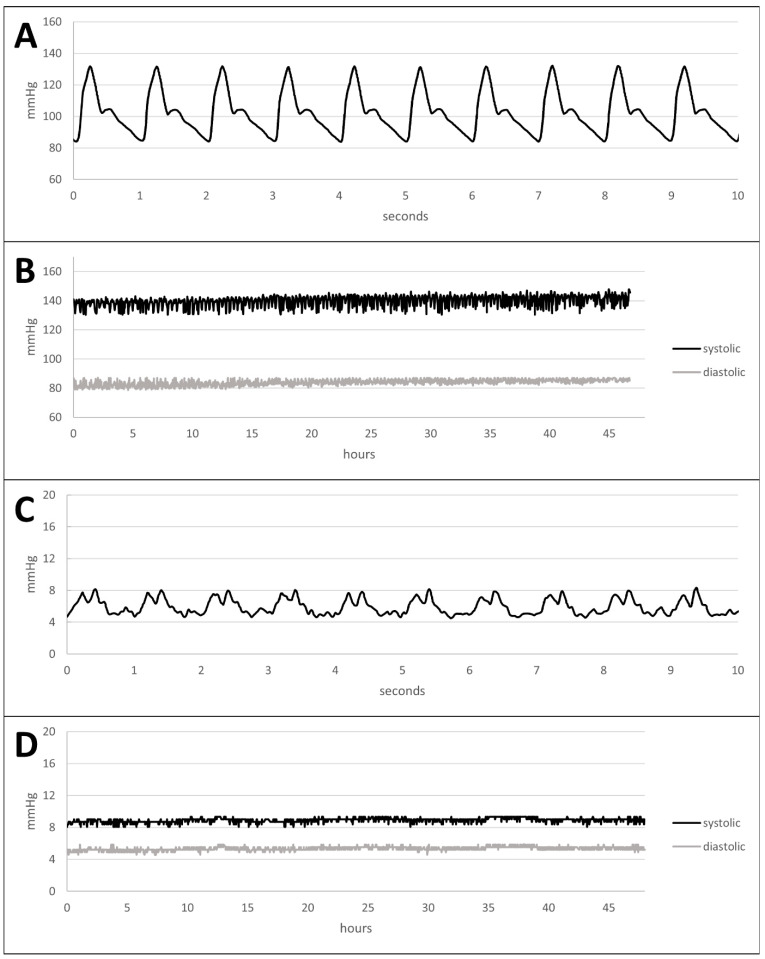
Pressure curve analysis. (**A**) Pressure curve monitored in the arterial circuit; (**B**) systolic (black) and diastolic (grey) pressures observed in the arterial circuit over 48 h. (**C**) Pressure curve monitored in the venous circuit; (**D**) systolic (black) and diastolic (grey) pressures observed in the venous circuit over 48 h.

Pressure and flow conditions were monitored continuously using the multisensor monitoring system. Here, the arterial pressure curve showed a dicrotic waveform with a steep upward slope up to a maximum pressure of approximately 140 mmHg followed by a dicrotic notch and a flatter downward slope (Figure 3A). In contrast to that, the venous pressure curve had a monocrotic morphology ranging from 5 mmHg to 8 mmHg (Figure 3C). Here, slight pressure variations and artifacts were present due to the lower pressure level. Both, arterial and venous pressures were constant over 48 h with slight variations between 130.4 mmHg and 147.9 mmHg systolic pressure and between 86.9 mmHg and 78.5 mmHg diastolic pressure for the arterial circuit (Figure 3B) and between 8.1 mmHg and 9.3 mmHg systolic pressure and between 5.8 mmHg and 4.6 mmHg diastolic pressure for the venous circuit (Figure 3D).

**Figure 4:**
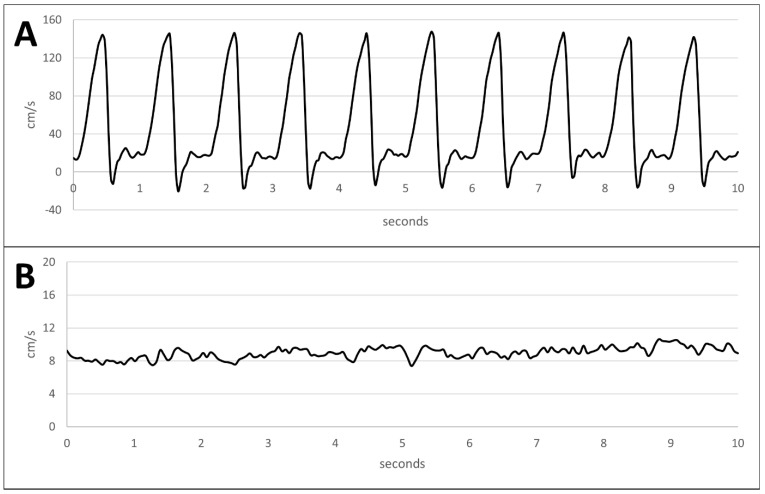
Flow curve analysis. (**A**) Flow curve monitored in the arterial circuit. (**B**) Flow curve monitored in the venous circuit.

The arterial flow curve was triphasic with antegrade phase in the early systole and a peak velocity of up to 140 cm/s followed by a short retrograde flow phase and an additional flatter antegrade phase (Figure 4A). The venous flow simulated in the perfusion system was continuous and static (Figure 4B).

### 3.3. Arterio-Venous Oxygen Partial Pressure Gradient

**Figure 5:**
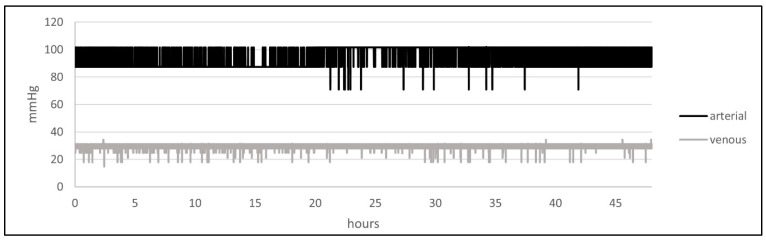
Arterial (black) and venous (grey) oxygen partial pressure monitored in the system over 48 h.

Automated oxygen supply to the arterial and venous reservoirs resulted in a mean arterial oxygen partial pressure of 94.3 mmHg ± 7.2 mmHg and a venous oxygen partial pressure of 28.9 mmHg ± 1.74 mmHg, respectively. Arterial and venous oxygen partial pressures remained constant with slight variations correlating with opening and closing of the solenoid valves over the course of 48 h (Figure 5).

### 3.4. Cellularized Matrix Perfusion

**Figure 6:**
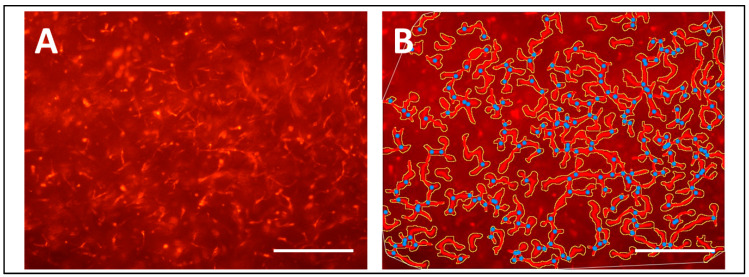
(**A**) Fluorescence microscopic view of the fibrin-based tissue matrix. Capillary tubes were visualized based on red fluorescent protein expression of human umbilical vein endothelial cells. (**B**) Angiotool analysis of the capillary network depicted in (**A**). Crossing points were marked by blue dots, capillary tubes were depicted in red, and outlines were marked in yellow. Scale bar = 100 µm.

For an initial proof-of-concept investigation, a cellularized fibrin-based matrix containing a co-culture of RFP-HUVECs and ASCs was performed. After 48 h of dynamic incubation in the modular arteriovenous perfusion system, capillary network formation through cellular self-assembly of HUVECs was observed in laser-scanning confocal fluorescence microscopy analysis (Figure 6). The tubular morphology of the capillary-like structures was confirmed via transmission electron microscopy showing the presence of a lumen built up by interconnected endothelial cells in a cross-section of a tube (Supplemental Figure S1). The pretest for direct microchannel perfusion revealed a shunt flow of 10.5 mL per minute for each microchannel, which remained constant over the test period of 48 h. Preliminary viability analysis revealed sustained cell viability of 92.5% after 48 h.

## 4. Discussion

### 4.1. Arteriovenous Perfusion System Technique

In vitro cardiovascular system emulators have been established to accurately simulate the biomechanical conditions of the human cardiovascular system on a desktop. In prior attempts, non-biological systems have been developed for the preclinical testing of mechanical support devices [14,15,23,24]. These systems, which in part offer a modular setup as well, facilitate highly accurate simulation of physiological and pathological pressure conditions in vitro. Some emulation systems even enable simultaneous simulation of arterial and venous mechanical conditions [13], which was also targeted in the present study. However, these systems do not facilitate the integration and stimulation of biological or bioartificial probes and are therefore unsuitable for tissue engineering approaches. In contrast, all components of the bioreactor perfusion system here were approved for cell culture and could be autoclaved at 121 °C and thereby sterilized. The placement of the entire system (except the pumps) within a standard cell-culture incubator with an adaptable oxygen supply facilitated the incubation of cellularized constructs with defined cell-culture conditions allowing for the use of standard cell culture media as perfusion media. The capillary tube formation observed in the fibrin-based matrix indicated suitability of the system for cell culture applications.

As an alternative approach, we and others have previously established numerous bioreactors and bioreactor perfusion systems that facilitate the implementation of biological probes [25,26]. Here, major differences in the accuracy of pressure and flow simulations can be noted. In some approaches, the simple simulation of sinusoid pressure curves with the respective target pressure [8,27] or systems optimized for the intended mechanical biological probe deformation without accounting for the physiological pressure curve morphology were used [16]. These systems usually only target physiological pressure curve simulation. Other systems were designed to emulate physiological pressure and flow conditions for bioartificial or biological blood vessels as accurately as possible [19,28]. While these systems allow for the implementation of biological probes and facilitate accurate emulation of the physiological pressure curves, they are targeted to emulate one specific arterial or venous anatomic site. For the physiological in vitro stimulation of the microvasculature, however, this is not sufficient as both the arterial and venous conditions must be simulated simultaneously in one system. In the present system, this was achieved by combining two perfusion circuits through the biological scaffold.

Furthermore, the physiological arteriovenous pressure gradient may also play a pivotal role in the generation of microvascular networks. Here, Wiliams et al. showed that directed capillary tube formation by cellular self-assembly, as well as vascular sprouting, may be augmented by hypoxic conditions [4]. Additionally, we previously demonstrated enhanced capillary network formation through the induction of pseudo-hypoxic conditions in vitro [29]. Thus, a comprehensive bioreactor perfusion system not only must take biomechanical factors into account but also needs to emulate physiological biochemical factors, especially the oxygen partial pressure gradient for the physiological stimulation of microvascular constructs. Here, the closed-loop automated oxygen supply unit implemented in both circuits facilitated the generation of a constant arteriovenous pressure gradient as it is present in the native arteriovenous microvasculature. The combination of biomechanical stimulation factors in form of physiological pressure and flow curves, on one hand, and the oxygen gradient, on the other hand, may further increase microvascular network formation.

Since no commercially available systems exist for this highly specific application, the arteriovenous perfusion system was established using a custom-built modular setup. As known from biopharmaceutical perfusion bioreactors, continuous online monitoring and data logging play an important role for in vitro perfusion systems [30]. In our system, this task was accomplished using a custom-built multisensory monitoring and data logging system, which was coordinated using a C++based code and a commercially available Arduino^®^ microcontroller platform. This facilitated parallel continuous monitoring of the different sensors for the parameter flow, pressure, and oxygen partial pressure. With that, monitoring and data logging were automated and simplified compared to previous systems using non-interacting sensor systems for different parameters within the perfusion system [19,31].

### 4.2. Pressure, Flow, and Oxygen Partial Pressure Emulation Compared to Physiological Values

The arterial pressure curve simulated in the perfusion system showed the typical dicrotic morphology that is present in native peripheral human arteries [5]; likewise, the triphasic flow profile of peripheral arteries was simulated accurately [7]. For venous flow and pressure, the characteristic monophasic pressure profile and static flow profile were simulated, as present in peripheral veins [7,32]. With an arterial oxygen partial pressure of 94.3 mmHg and a venous partial pressure of 28.9 mmHg, oxygen partial pressure values were within the physiological limits for the arterial and venous circulation [33,34].

### 4.3. Microvascular Network Formation

Incubation in the dynamic bioreactor perfusion system facilitated the generation of a dense capillary network and thus reproduced the capillary network formation results achieved in a preliminary study using static perfusion [20]. Furthermore, the seeding ratio of HUVECs and ASCs of 1:0.5, which was previously shown to facilitate capillary network formation in fibrin gels [35], was shown to be suitable for capillary tube formation under dynamic perfusion as well. While this serves as a proof of concept for the applicability of the bioreactor perfusion system for capillary cell culture, further analyses of the influence of arteriovenous perfusion on microvascular angiogenesis are still pending, whereby the response of an intimal endothelial monolayer in larger bioartificial vessels and microchannels has already been described before [9,18].

### 4.4. Limitations

While the bioreactor perfusion system presented here facilitates multi-sensor monitoring in one device, closed-loop adaptions were only facilitated for the oxygen partial pressure regulation by adjustable solenoid valves, while the pumps and resistors had to be adjusted manually. Furthermore, the potential benefits of different physiological perfusion models simulated in the system have not been investigated yet as this study focused on the technical aspects of the arteriovenous perfusion system.

### 4.5. Outlook and Future Applications

Future potential applications of the arteriovenous perfusion system presented here are twofold: on one hand, the system can be used to optimize in vitro culture techniques for tissue engineering approaches targeting the generation of complex multi-scale vascular networks and the generation of larger three-dimensional bioartificial tissues. On the other hand, the system can potentially be used to generate an in vitro platform for disease modeling or drug testing on native or bioartificial tissues stimulated under physiological conditions simulated in the arteriovenous perfusion system. In initial analyses, the influence of arterial, venous, and arteriovenous perfusion on the formation of the microvascular network will be investigated.

## Data Availability

Data used for this study are available from the corresponding author upon request.

## Supplementary Materials

The following supporting information can be downloaded at: https://www.mdpi.com/article/10.3390/bioengineering11111147/s1, Figure S1: Transmission electron microscopy image of a cross section of a capillary-like tube. Arrows indicate Zonulae occludentes between the cells forming the lumen. Scale bar = 5 μm.
