# Supplementary figures and images for: An Arteriovenous Bioreactor Perfusion System for Physiological In Vitro Culture of Complex Vascularized Tissue Constructs
Source: Bioengineering (Basel). 2024 Nov 14;11(11):1147. doi: 10.3390/bioengineering11111147 (PMC11591738; doi:10.3390/bioengineering11111147)

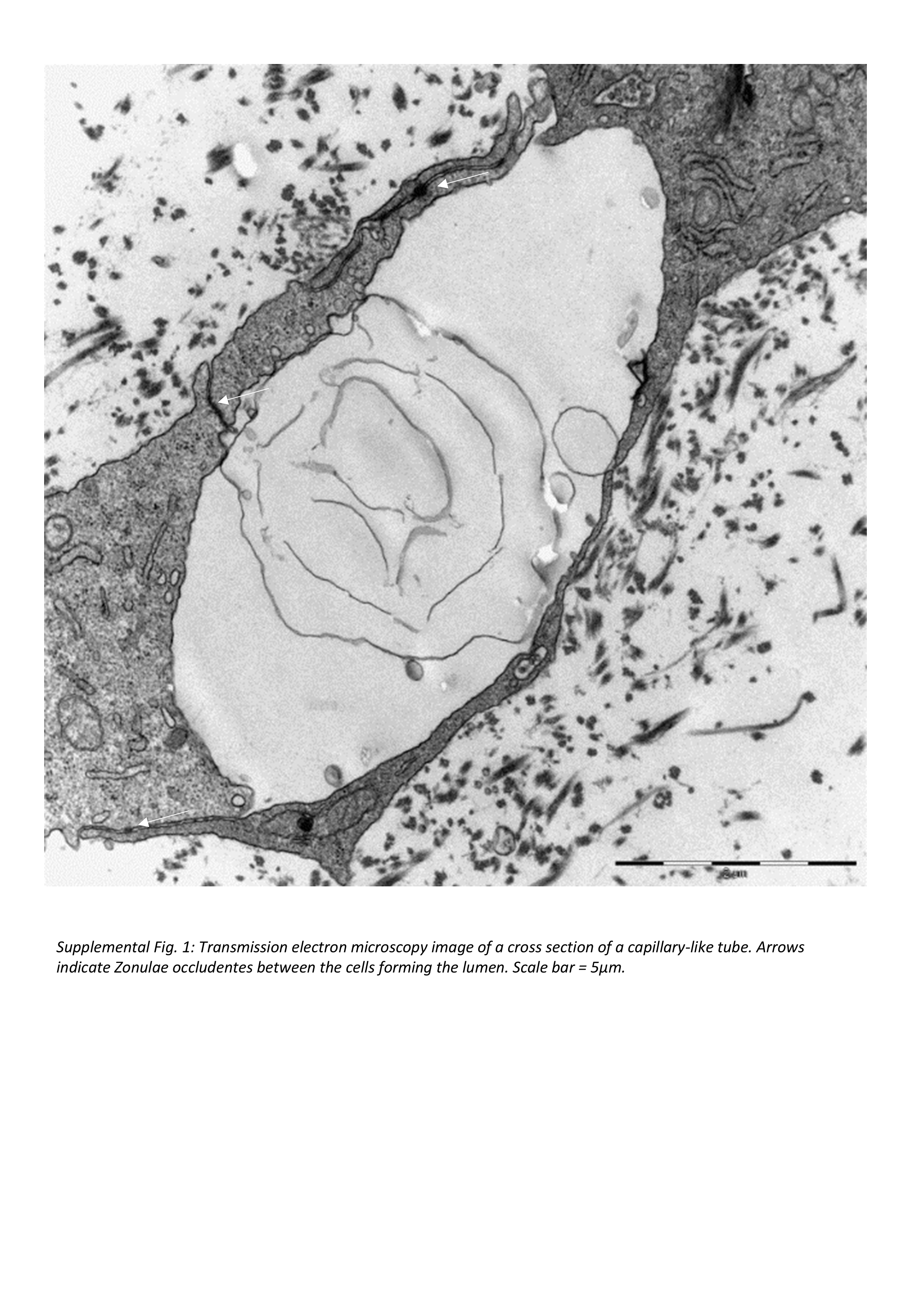

Supplement: Supplementary file 1 [file bioengineering-11-01147-s001.zip › bioengineering-3288095-supplementary.tif]
